# An evaluation of African animal trypanosomiasis control strategies in remote communities of Eastern Zambia

**DOI:** 10.1017/S0031182024001070

**Published:** 2024-09

**Authors:** Gloria M. Mulenga, Kalinga Chilongo, Chrisborn Mubamba, Bruce Gummow

**Affiliations:** 1 Department of Veterinary Services, Kakumbi Tsetse and Trypanosomiasis Research Station, Airport Road, Mfuwe, Zambia; 2 Department of Veterinary Services, Ministry of Fisheries and Livestock, Lusaka, Zambia; 3 College of Public Health Medical and Veterinary Sciences, James Cook University, Townsville, Australia; 4 Faculty of Veterinary Science, University of Pretoria, Pretoria, South Africa

**Keywords:** African trypanosomiasis, cattle, incidence, prevalence, Zambia

## Abstract

Communities living in African animal trypanosomiasis (AAT) endemic areas of Zambia use several control strategies to protect their livestock from the devastating effects of trypanosomiasis. Several studies have reported the effectiveness of trypanosomiasis control strategies based on retrospective data. In this study, we assessed incidence rates of AAT in cattle (*n* = 227) using a prospective cohort study comprising 4 treatment groups, i.e., Diminazene aceturate, Isometamidium chloride, Cyfluthrin pour-on and Cypermethrin treated targets. The study was conducted in Mambwe district in Eastern Zambia between February 2019 and March 2020. The endemic prevalence of AAT for each group was determined using ITS-PCR prior to application of treatments. High endemic trypanosome pre-treatment rates were found in all Groups (Diminazene aceturate (61%), Isometamidium chloride (48%), Cyfluthrin pour-on (87%) and Cypermethrin targets (72%)). The overall apparent prevalence for the Mambwe district was 67% (152/227) and true prevalence at 95%CI was 63–71%. Once treatments were implemented, 12 monthly follow-ups were conducted. The average monthly incidence rates without standardization recorded: Diminazene aceturate (67%) Isometamidium chloride (35%), Cyfluthrin pour-on (55%) and Cypermethrin targets (61%). Incidence rates were standardized considering the endemic level of disease for each Group and the average standardized monthly incidence rate in the Diminazene aceturate Group was 7%; the Isometamidium chloride Group −13%; the Cyfluthrin Group −26%; and the Cypermethrin target Group, −17%. All Groups showed a decrease in incidence of AAT over the period of the study with the Cyfluthrin group showing to be the most effective in reducing AAT incidence in cattle.

## Graphical abstract

**Figure:**
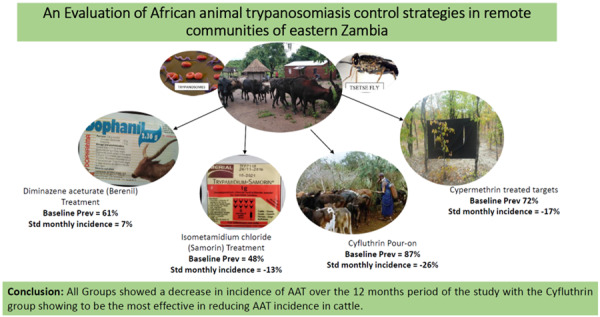

## Introduction

African animal trypanosomiasis (AAT), also known as nagana, is a major constraint to livestock production in settled parts of tropical Africa (Swallow, 2000; Muhanguzi *et al*., 2017). In Africa alone, 50 million head of cattle are at risk of the disease. Direct losses are estimated at USD 1.2 billion per year and about US$4.5 billion for overall agriculture production (Franco *et al*., 2020; WHO, 2022). Cattle dominate the livestock sector in Zambia, both among the commercial and traditional farmers in Zambia. According to the 2018 livestock and aquaculture Census, the livestock population in Zambia stood at 3.7 million cattle, 3.5 million goats, 170 thousand sheep and 1.1 million pigs (Ministry of Livestock and Fisheries (MFL) and Central statistics Office (CSO), 2019). The Zambian agricultural industry has not been spared from the devastating effects of AAT. Over 60% of the country's cattle population is under threat from trypanosomiasis (MFL, 2017). The prevalence of trypanosomiasis in livestock and particularly in cattle ranges between 1% and 90% depending on the location (Simukoko *et al*., 2011; MFL, 2017; Mulenga *et al*., 2021).

Livestock rearing in tsetse infested regions has been restricted due to the drastic effects of trypanosomiasis. The disease is associated with very serious economic consequences, such as reduced productivity and fertility, livestock death, increased abortion, and high treatment costs (Shaw *et al*., 2014, 2015). Most livestock owners in tsetse infested areas have resorted to extensive use of various chemotherapeutic treatments to combat the disease (Van den Bossche and Delespaux, 2011; Mulenga *et al*., 2020).

Common tsetse and trypanosomiasis control methods employed in Zambia, include the use of odour baited Cypermethrin targets, animal treatment with trypanocides, and dipping (Mulenga *et al*., 2020). The use of odour baited Cypermethrin treated targets involves a suspended screen of blue/black or black cloth (tsetse target) impregnated with insecticide. Tsetse flies are attracted to the screen by the odour bait and land on the black segment, where they collect a lethal dose of the insecticide on contact and later succumb. Significant catches of the Morsitans group of tsetse are recorded when baited with attractants that mimic the odours of the natural hosts, while the Palpalis tsetse group catch can be increased through baiting with both natural and synthetic odours. Formulated blends (1:4:8) of 3-*n*-propylphenol (P), octanol (O), and *p*-cresol (C), together with separately released acetone (A) (collectively referred to as POCA) have shown enhanced attraction to *Glossina morsitan morsitans* or *G*. *pallidipes* (Rayaisse *et al*., 2010; Mireji *et al*., 2022).

The use of insecticides was engineered for use in several ways such as ground spraying, aerial spraying, sequential aerosol technique, or in more localized areas using hand-held or vehicle-mounted fogging machines, and artificial and live-bait techniques (Vale *et al*., 2015; Percoma *et al*., 2018; Lord *et al*., 2020). Treating cattle with insecticide and deploying insecticide treated targets are more affordable and sustainable interventions employed by livestock farmers compared to complex alternatives such as aerial spraying or sterile insect technique. However, treating cattle with insecticide may not only control tsetse but also control tick-borne diseases, while insecticide treated targets will only reduce trypanosomiasis incidence cases in livestock (Hargrove, 2004; Vale *et al*., 2015). Chemotherapy and chemoprophylaxis in animals are the commonly used options in the control of AAT by individual livestock owners. They are based on screening/inspection and treatment of livestock hosts found positive for trypanosomiasis. In Africa, the most commonly used drugs for treatment of AAT are Berenil (Diminazene aceturate® Dopharma Inter. Raamsodnksveer, The Netherlands) and Samorin (Isometamidium chloride® Merial Ltd, Lyon-France) (Delespaux *et al*., 2002; Mungube *et al*., 2012; Mbewe *et al*., 2015) Diminazene aceturate is usually used as a curative drug for the treatment of AAT whilst Isometamidium chloride is used as a prophylaxis (FAO, 2002). The two drugs have been reported to be very effective against strains of *Trypanosoma congolense* and *T. vivax*. The interval between successive administrations of a prophylactic drug will vary between different drugs and according to the level of trypanosome challenge the animals are exposed to. Protection with Isometamidium chloride treatment has been shown experimentally to be between 3 and 6 months depending on exposure rates (Mungube *et al*., 2012; Fyfe *et al*., 2017; Hamill *et al*., 2017; Mulenga *et al*., 2017). Prolonged and exclusive use of both trypanocides and insecticides could however, lead to drug resistance (WHO, 2021).

Choosing an effective AAT control method for trypanosomiasis affected livestock farmers is critical and is influenced by the availability and affordability of the control method (Geerts *et al*., 2001). Most livestock farmers reportedly, treat their livestock based their own visual inspection of animals, in the absence of a veterinary professional (Kasozi *et al*., 2023). Limited diagnostic capacities and professional personnel has contributed negatively to the control and management of emerging and remerging diseases, including trypanosomiasis in Sub-Saharan Africa (Mwanakasale *et al*., 2013; Mulenga *et al*., 2015). Livestock farmers in trypanosomiasis endemic areas in Zambia continue to spend large amounts of money and resources to protect their animals from the devastating effects of tsetse and trypanosomiasis. Most assessments on the control of tsetse and trypanosomiasis have been based on retrospective prevalence data (Tofthagen, 2012; FAO, 2017; Meyer *et al*., 2018; Kizza *et al*., 2022). The data for such studies is often not collected with the specific purpose of comparing control strategies, thus introducing potential bias into the results, and the use of prevalence data precludes an accurate estimation of the risk of an animal becoming infected (Thrusfield, 2005). A prospective study can be designed around the specific objectives of the study and reduce the effects of recall bias and confounding as well as measure the real time effect of a control strategy. Such studies can generate incidence data that enables far more powerful statistical tests and measures of association to be used to confirm the significance of the findings (Thrusfield, 2005). In this paper, using a prospective cohort study, we compared incidence rates of AAT in cattle under three tsetse and trypanosomiasis control interventions that are commonly used in Zambia, i.e., Chemotherapy (Isometamidium chloride and Diminazene aceturate) Cyfluthrin pour-on and Cypermethrin treated targets (Delespaux *et al*., 2002; Mbewe *et al*., 2015; Meyer *et al*., 2016)

## Materials and methods

### Study area and animal recruitment

**Figure 1.**
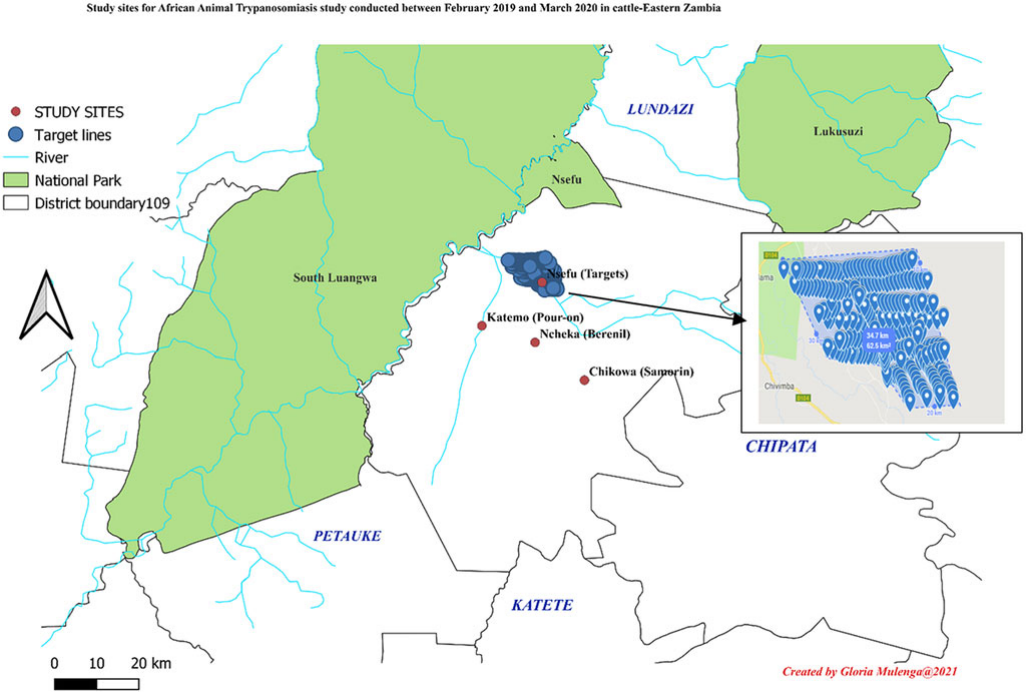
Map showing study sites for the 4 treatment groups (Insert showing area deployed with targets).

The study was conducted in Mambwe district, eastern Zambia. The district was purposively selected because it is highly tsetse infested and has a high prevalence of bovine trypanosomiasis (Laohasinnarong *et al*., 2015; Mulenga *et al*., 2022). Located along the Luangwa River basin, Mambwe district covers an area of 4,480 km^2^ and includes part of the South Luangwa National Park (Fig. 1). Mambwe district has a population of 92 445 people translated into 18 489 households. Most of the local community rely on wildlife tourism and small-scale farming for their livelihoods (Zambia Central statistics data 2015). Communal animal grazing is a common practice for livestock farmers in the area. Tsetse transmitted trypanosomiasis is one of the major diseases occurring in Mambwe district. Affected communities have employed several control methods to combat the disease, which include the use of odour baited targets, chemotherapy, and dipping (MFL, 2017). Livestock farmers were recruited by field veterinary assistants in February 2019, based on their years of experience in cattle keeping and their willingness to participate. Written informed consent from each farmer was required prior to their participation in the survey. Taking part in the study was completely voluntary and farmers understood that they were free to withdraw from the study without penalty. All participating farmers were supplied with an information sheet and consent form that had been approved by the James Cook University Human Ethics committee (ID A2498) (Supplementary data attached).

Farmers and their animals were only recruited after details on the information sheet had been read to them and consent forms signed. Only adults older than 19 years of age (of sound mind) were allowed to participate in the study. All cattle included in the study were ear tagged with a unique number for easy identification. Both young (weaned from their mothers) and adult animals were included in the study. Age was determined by a veterinarian who was part of the research team using the dentition method (Dyce *et al*., 2009).

### Study design

A prospective cohort study of AAT incidence rates between February 2019 and March 2020 in cattle was carried out using 3 control options commonly used to control AAT in Eastern Zambia. The chemotherapy option was divided into 2 separate groups i.e. Diminazene aceturate and Isometamidium chloride groups, therefore creating 4 treatment groups for the study. Four cohort groups (herds) were created corresponding to each treatment group and followed monthly. The 4 cohort groups were in 4 separate trail sites within the same district of Mambwe and approximately 50 km of each other to prevent carry over treatment effects from one trial site to another. The 4 trial site crush pens (Nsefu, Katemo, Chikowa, Ncheka) (Fig. 1) were purposively selected based on the commonality between the sites. The sites were similar with respect to climate, livestock, and human populations within the wildlife interface areas (man and his livestock are casual intruders) where the likelihood of tsetse bites by infected flies was high. All 4 sites had similar temperature and rainfall patterns. Average daily rainfall and temperatures were recorded and collated in 4-week periods corresponding with those of the chemotherapy treatments. All 4 groups were comprised of the local ‘Agoni’ cattle breed with similar age and sex patterns across the herds. The husbandry practices, nutrition, biosecurity, and use of labour was similar for all 4 groups and followed standard local cattle keeping practices used by all villages in the district. Hence, the trial sites and cattle herds at each site were largely matched, which was an important part of the study design.

### Sample size

Using an estimated prevalence of AAT of 35% based on routine AAT surveillance conducted by the Department of Veterinary Services in non-treated areas of Mambwe district (Kakumbi tsetse and trypanosomiasis research station 2017) and previously conducted studies (Mubamba *et al*., 2011), and assuming the treatments in the study would decrease the prevalence to an average of 10%, then 43 cattle were needed in each treatment group to show a reduction in AAT prevalence of 25% between an untreated and treated group (Hulley *et al*., 2013).A total of 227 cattle were enrolled (Diminazene aceturate group 64, Isometamidium chloride group 48, Cyfluthrin pour-on 47, and Cypermethrin target group 68). The cattle for each group (trial area) were selected as herds of cattle belonging to individual farmers that were farming in the area where the trial was being conducted. The number of owners selected and the respective number of cattle each owner had at each trial site is shown in Table 1.

**Table 1.** Demographic data showing the number of cattle owners and cattle per trial group, Mambwe district, 2019

	No. of livestock farmers	No. of cattle herds	Cattle enrolled	Female cattle	Male cattle	Young cattle	Adult cattle
Diminazene aceturate	49	3	64	31	33	12	52
Isometamidium chloride	47	3	48	24	24	14	34
Cyfluthrin Pour-on	45	17	47	22	26	8	40
Cypermethrin target	52	11	68	30	37	9	58
Total infections on ITS-PCR				40	43	10	73
Totals	193	34	227				

*Note*: Total number of infections is the cumulative number of infections that occurred throughout the study period.

Animals previously infected and re-infected during the study were counted as new infections at every sampling time.

Farmers at each trial site made use of communal grazing and cattle from owners (including those not enrolled in the study but belonging to the same site) co-mingled and moved within a communal herd and rarely as individual herds. Hence, cattle at each trial site should be viewed as a communal herd and a single body of cattle and not as clusters based on the owner. The cattle farmers in the respective trial areas were purposively selected based on their willingness to participate. Written informed consent from each farmer was required prior to their participation in the study.

### Treatment groups

The selected trial sites were allocated to 4 treatment groups i.e., Diminazene aceturate treatment, Isometamidium chloride treatment, Cyfluthrin pour-on and Cypermethrin targets. Two weeks before initiating the intervention strategies, all animals were treated with Diminazene aceturate (3.5 mg kg^−1^ b.w. deep intramuscular injection) (FAO, 2002; Mungube *et al*., 2012) to clear any existing trypanosomes. The assumption was therefore that cattle were free of trypanosomes at the start of the study. Due to ethical issues, at every monthly sampling, trypanosome infected animals from each treatment group were treated with Diminazene aceturate (within 24hrs of testing positive on microscopy) at the same dose as described above, with the Diminazene aceturate treatment being constant for all groups. The assumption was that Diminazene aceturate would sterilize trypanosome infections and that each new month was regarded as a new trial. All cattle in the 4 groups were therefore, assumed to be cleared of trypanosome infection at the start of each month, allowing for a monthly incidence rate to be calculated. Monthly incidence rates were calculated based on polymerase chain reaction (PCR) results.

#### Group 1-diminazene aceturate treatment

At every monthly sampling, all cattle found positive for trypanosomes during the month of sampling were treated with Diminazene aceturate at a dose as described above. This group received no other treatments.

#### Group 2-isometamidium chloride treatment

Cattle in this group were treated every 12 weeks with Isometamidium chloride by deep intramuscular injection at a dose of 0.5 mg kg^−1^ in 2% solution as described by Mungube *et al*. (2012) and recommended by FAO (2002) guidelines.

#### Group 3-cyfluthrin pour-on

The Cyfluthrin pour-on group were treated with Cyfluthrin (Amipor-Virbac Pty, Ltd, South Africa) pour-on every 8 weeks at 15 mL/100 kg. Insecticide application was restricted to belly and legs (biting sites for tsetse). Restricted application reduces cattle dung contamination and costs by 40% as compared to full body application (Torr *et al*., 2007a; 2007b; Vale *et al*., 2015).

#### Group 4-cypermethrin targets

Insecticide treated black cloths (Cypermethrin 1:9 concentrate) baited with Butanone (50 mg h^−1^) and 1-octen-3-ol (0.5 mg h^−1^) (AVIMA-Pty-Ltd, South Africa) were deployed in the area infested with *G. morsitans* group of tsetse flies (Torr 2007), where animals in the target group grazed. In total, 300 insecticide targets were deployed within a 62.5 km^2^ area which was slightly above the minimum size (60 km^2^) required for deployment (MFL, communications). Deployment was done at 250 m intervals over a linear distance with focus on paths that animals use for grazing or drinking water. Four Cypermethrin targets were deployed per km^2^. The width of deployed Cypermethrin targets ranged between 2–5 km (Fig. 1). GPS coordinates were recorded for all Cypermethrin targets deployed (Kamba Mebourou *et al*., 2020; Rayaisse *et al*., 2020). However, the use of Cypermethrin targets has been associated with vandalism and destruction by wild animals. To overcome such limitations, locals were involved in the deployment (to create awareness), monitoring and maintenance conducted every 3 months (MFL, communications).

### Sampling and treatment procedure

At the beginning of the study, all enrolled animals were screened to determine their baseline prevalence rates of trypanosomiasis prior to the implementation of the treatments. Prevalence was defined as the number of AAT positive cattle in a group divided by the total number of cattle in that group. An animal was regarded positive if trypanosomes were identified on ITS-PCR. The 95% confidence interval for the true prevalence was calculated using the standard error according to Thrusfield (2005). A specificity of 72.2%; and sensitivity of 77.5%, based on Mulenga *et al*.'s (2021) work, was factored into the true prevalence calculation.

All cattle were screened for trypanosomes using blood samples collected monthly over a period of 12 months between 2019 and 2020. Blood samples were collected by puncturing animal ear veins with blood lancets and collected using two micro capillary tubes containing an anticoagulant (Heparin). For each animal, about 200 μL of whole blood collected from the first capillary tube was placed on a labelled FTA® card and left to air dry out of direct sunlight. All collected samples on FTA® cards were packed in zip-locked storage bags containing silica gel and transported to the laboratory where they were stored at ambient temperature for further processing on ITS-PCR. Blood from the second capillary tube was used to make blood slide thin and thick smears for further microscopic examination after staining with Giemsa solution (Katsidzira and Fana, 2010). Supplementary data for each animal was recorded (breed, sex, age, location, and date of sampling) and categorised as young or adult. GPS coordinates were recorded for each sampling site.

Trypanosome infection for the four treatments groups was determined using both microscopy and PCR (Cox *et al*., 2010; Ahmed *et al*., 2013; Mulenga *et al*., 2021). Being a simple and quick test, infections detected by microscopy were used to treat infected animals during monthly follow-ups. Infected animals were treated within 24 hours using Diminazene aceturate at the dose described above. Diagnosis was further improved using PCR to calculate monthly incidence rates (Mungube *et al*., 2012; Hassan-Kadle *et al*., 2020; Mulenga *et al*., 2021). Sensitivity of the diagnostic tests used has been discussed in a separate paper Mulenga *et al*. (2021).

### Laboratory analysis

#### Sample preparation

Samples collected on FTA® cards were prepared by puncturing two 3 mm diameter discs from each card using a Harris micro-punch Tool. The discs were placed in 1.5 mL sterile tubes accordingly and labelled. The discs were then washed twice in 100 μL of Whatman purification reagent for 15 minutes followed by 2 washes in 100 μL of 1x-concentrate TE buffer for 15 minutes to remove any residual Whatman purification reagent. The discs were transferred to labelled 100 μL tubes and allowed to dry at room temperature. Finally, after the discs had dried, DNA was eluted using a Chelex 100® elution protocol. The eluted DNA was stored at 4°C for use within 12 hours and at −20°C for use after 12 hours (Morrison *et al*., 2007; Anderson *et al*., 2011; Mulenga *et al*., 2021). ITS-PCR was performed as described by Njiru *et al*. (2005). At the same time at the laboratory, thin (fixed with methanol) and thick smears made in the field were stained with 10% Giemsa solution for 30 minutes and later examined microscopically at 100× oil immersion objective lens (1000× magnification) for the presence of trypanosomes (FAO, 2002; Marcotty *et al*., 2008; Mulenga *et al*., 2021). Microscopy results were available to livestock farmers within 24hrs prior to sampling to allow for treatment of animals found positive for trypanosomes.

#### Disease incidence

Monthly laboratory positive samples on ITS-PCR were used to calculate incidence rates. The endemic trypanosome apparent prevalence rates of each treatment site were recorded prior to initiation of the control treatments and used as a baseline for the disease. Trypanosomiasis incidence rates for the four groups over the entire period of the study were standardized by subtracting the background pre-treatment prevalence from each monthly post-treatment incidence rate. This was done to account for possible differences in exposure rates between sites. Analysis of variance (ANOVA) was used to determine whether there were any statistically significant differences in mean standardized incidence rates (mean proportion rates) between the four treatment groups. In a post hoc analysis, the Bonferroni (All-Pairwise) Multiple Comparison Test was used to determine differences in means between the pairs of the treatment groups (*α* = 0.05, DF = 44, MSE = 0.046, Critical Value = 2.76) and Scheffe's Multiple Comparison Test (*α* = 0.05, DF = 44, MSE = 0.046, Critical Value = 2.91).

## Results

### Trypanosomiasis prevalence and incidence

Baseline trypanosome apparent prevalence rates (recorded before interventions) for the treatment groups and their corresponding 95% confidence intervals for true prevalence factoring in the sensitivity and specificity of the ITS-PCR test were as follows: Diminazene aceturate treatment 61%, (39/64), (95% CI for true prevalence = 53–69%), Isometamidium chloride treatment 48%, (23/48), (95% CI for true prevalence = 38–57%), Cyfluthrin pour-on 87%, (41/47), (95% CI for true prevalence = 81–84%), and Cypermethrin targets 72%%, (49/68), (95%CI for true prevalence = 65–79%). The overall apparent prevalence for Mambwe district was 67% (152/227) and the 95%CI for true prevalence = 63–71%.

**Figure 2.**
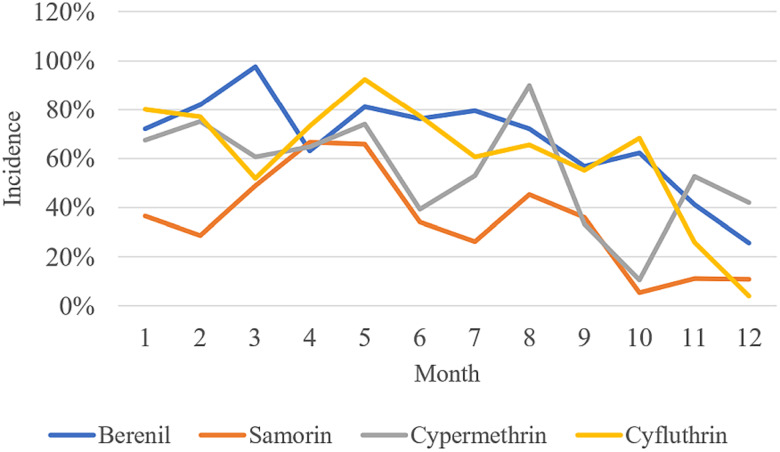
Change in crude incidence rates between treatment groups during the study period conducted in the Luangwa Valley of Eastern Zambia between the 2019 and 2020.

**Figure 3.**
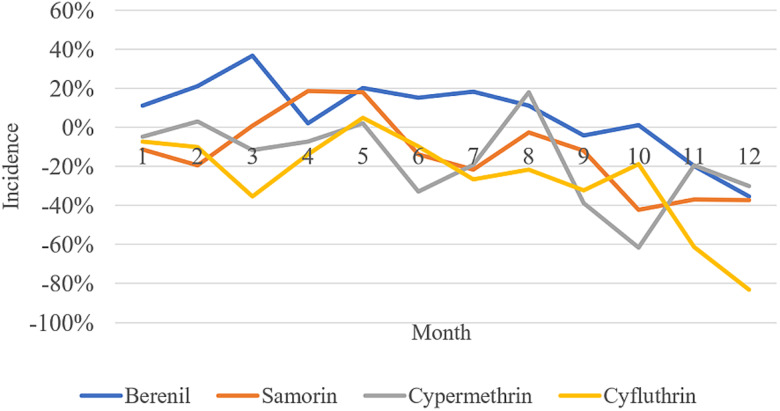
Change in standardized incidence rates between treatment groups during the study period conducted in the Luangwa Valley of Eastern Zambia between the 2019 and 2020.

The average monthly incidence rates without standardization over the 12-month period after initiation of the treatments were as follows: Diminazene aceturate treatment = 67%; Isometamidium chloride treatment = 35%, Cyfluthrin pour-on = 55% and Cypermethrin targets = 61%. Individual crude monthly incidences are shown in Fig. 2. When standardized by subtracting the endemic prevalence, the average standardized incidence rates were: Diminazene aceturate treatment = 7%; Isometamidium chloride treatment = −13%, Cyfluthrin pour-on = −26% and Cypermethrin targets = −17%. The monthly standardized incidence rates are show in Fig. 3. The results indicated a significant drop in monthly incidence for all the treatment groups over the study period (Fig. 3).

### Statistical significance between treatment groups

Results showed significant differences in both crude and standardized mean monthly incidence rates between the treatment groups (*p* value <0.01, *F* value = 16.181) when compared using ANOVA. The mean monthly standardized incidence rates were then compared, using the Bonferroni (all-pairwise) multiple caparison test (*α* = 0.05, DF = 44, MSE = 0.046, Critical Value = 2.76) and Scheffe's Multiple Comparison Test (*α* = 0.05, DF = 44, MSE = 0.046, Critical Value = 2.91).

Results indicated differences between group means as shown in Table 2.

**Table 2.** Standardized incidence rates when compared between groups

Group	Count	Mean	Different from groups
Diminazene aceturate	12	0.65	Cyfluthrin
Isometamidium chloride	12	−.013	
Cypermethrin targets	12	−0.17	
Cyfluthrin	12	−0.26	Diminazene aceturate

The Duncan's Multiple Comparison Test, which is less conservative found significant differences between Diminazene aceturate and all the other treatment groups (*α* = 0.05, DF = 44, MSE = 0.046).

### Weather

During the 13 monthly treatment period, the mean rainfall ranged between 0.00 mm to 288 mm while mean minimum temperatures ranged between 11°C and 21°C, and mean maximum temperatures ranged between 31°C and 39°C. High temperatures were reported in the middle of the rainy season at the beginning of the study, while an increase in rainfall and temperature were later seen at the start of the next rainy season from month 11 to 13.

## Discussion

This is one of few studies to report the point prevalence of AAT in village cattle communally farmed in Mambwe district of Eastern Zambia, which was higher than previously reported (Mubamba *et al*., 2011). This may have been due to the study beginning in the rainy warm season which provided a favourable environment for tsetse populations, and location of the study site closer to the game reserve, which acts as a reservoir for tsetse flies and trypanosomiasis, therefore predisposing cattle to AAT infection. In addition, many previous studies only used microscopy as a means of determining prevalence, whereas this study also used PCR, which is more sensitive. Challenges in the diagnostic performance of the parasitological and molecular tests used in this study have been discussed in a previous article by the authors (Mulenga *et al*., 2021)

This study is one of few studies that have used a cohort study design to assess the incidence of AAT in cattle under different treatment strategies. The advantage of incidence data is that it allows a more accurate assessment of the absolute risk of infection, and it is clear from the results that all the treatment strategies evaluated did reduce the risk of mortalities. The cohort study design also provided a more accurate way of comparing the efficacy of the drugs in the different treatment groups.

The results indicated that the vector control groups (Cypermethrin targets and Cyfluthrin pour-on) were the more effective in reducing trypanosome incidence than the parasite control groups (Isometamidium chloride treatment and Diminazene aceturate treatment). Our findings were in agreement with observations made in other studies (Hamill *et al*., 2017; Kamba Mebourou *et al*., 2020; Lord *et al*., 2020; Rayaisse *et al*., 2020). Baseline trypanosome prevalence rates were high for all four treatment groups compared to incidence rates after initiation of treatments. Monthly trypanosome incidence rates however, fluctuated over time and reduced towards the end of the study period indicating the effectiveness of the control strategies employed. High rainfall and temperatures were experienced at the time the baseline survey was conducted and towards the end of the survey. The wet-warm weather has been reported to favour tsetse population growth and tsetse movements resulting in increased transmission and infection rates in animals (Van den Bossche *et al*., 2010; Van den Bossche and Delespaux, 2011).

Animal protection from the vector control method is dependent on the chemical residual effects and technical issues related to the use of the control method (Tekle *et al*., 2018; Kamba Mebourou *et al*., 2020). The high toxicity and long residual effect of Cypermethrin insecticide in the black target materials allowed for long periods of effective vector control. This resulted in the reduction in trypanosome incidence rates in the group over time. Residual effects of Cypermethrin have been reported as effective for a period of 12 months, after which efficacy starts to reduce. Annual re-deployment of Cypermethrin targets is therefore recommended. The use of insecticide treated Cypermethrin targets has received much attention as one of the leading treatments effective in reducing tsetse populations, which in turn reduces trypanosome case detection in man and livestock (Courtin *et al*., 2015; Kamba Mebourou *et al*., 2020; Rayaisse *et al*., 2020). The control of tsetse populations using odour baited Cypermethrin targets has been considered more effective as a suppression method and for protecting small, localized farming communities. Several technical issues have however, been associated with Cypermethrin targets, which include theft, vandalism, and maintenance challenges (Vreysen *et al*., 2013).

Cattle are natural hosts for the tsetse vector, thus, baiting them with insecticide is a more logical method to protect them from tsetse bites. The application of Cyfluthrin pour-on on cattle not only offers protection against tsetse bites, but also provides control for ticks thus improving animal health and increasing meat and milk productivity to ensure food security (Kamau *et al*., 2000). Cyfluthrin pour-on is convenient and less demanding than other vector control methods. Cyfluthrin pour-on application has limited adverse effects on the environment, thus making the method, environmentally friendly. Challenges in the use of Cyfluthrin pour-on include among others, the costs associated with the treatment frequency, reduced farmer motivation to adhere to the Cyfluthrin pour-on application schedule, and risk of re-infections from other untreated animal disease reservoirs in the area (Kamau *et al*., 2000; Vreysen *et al*., 2013).

While several trypanosomiasis control methods target the tsetse vector, treatment of trypanosome infected animals with trypanocides continue to be the most widely applied control methods (Percoma *et al*., 2018). Our study employed two treatments which target the trypanosome parasite in livestock i.e., Diminazene aceturate treatment and Isometamidium chloride treatment. In the Diminazene aceturate treatment and Isometamidium chloride treatment groups, trypanocides are administered directly to the targeted animals and they are most effective in reducing parasite levels in the absence of drug resistance (Fyfe *et al*., 2017; Mulandane *et al*., 2018). Our findings showed that the Isometamidium chloride treatment group was more effective in reducing trypanosome incidence compared to the Diminazene aceturate treatment group. Increased infections in the Isometamidium chloride treatment group were however, observed during times when scheduled treatments were due. This may have been due to the diminished levels of prophylaxis around that time (Tekle *et al*., 2018). Reports from the field indicate that the period of protection is reduced by high tsetse challenge. Experimental evidence, however, does not confirm this common observation. Even in the absence of tsetse, infected livestock can trigger infections in other livestock and humans *via* other vectors like tabanids (Van den Bossche *et al*., 2010; Baldacchino *et al*., 2014). Such findings indicate that treatment of trypanosome infected livestock using trypanocides can be used to reduce the risk of trypanosome transmission between livestock and man and may also limit spill overs from wildlife (Hamill *et al*., 2017; Meisner *et al*., 2019; Lord *et al*., 2020; Mulenga *et al*., 2021).

In relation with findings of this study, Mulenga (2023) used a stochastic partial budget analysis model (Rushton, 2009; Lowa, 2018) to assess the financial returns of 4 tsetse and trypanosomiasis control methods (Diminazene aceturate treatment, Isometamidium chloride treatment, Cyfluthrin pour-on and Cypermethrin targets) through a prospective cohort study of AAT incidence in cattle. Results from the partial budget analysis showed that all 4 treatment groups yielded a positive financial net return but varying net values. The median net returns from the distribution functions as calculated in the partial budget showed that the Isometamidium chloride treatment group had the greatest return followed by the Cypermethrin target group and the Diminazene aceturate treatment group. The Cyfluthrin pour-on group showed the lowest treatment cost and yielded the lowest return. The model was most sensitive to additional returns due to increased births from reduced mortality and foregone returns due to cattle deaths (Mulenga, 2023). While several studies conducted on the costs of controlling trypanosomiasis have focused on the cost and benefit analysis (FAO, 2017; Meyer *et al*., 2018; Kizza *et al*., 2022), the study by Mulenga (2023) was the first to report the use of a partial budget analysis with a focus only on changes in farm income and expenses that would result from the implementation of specific alternatives. Based on incidence results alone Cyfluthrin pour-on would most probably be the drug of choice for livestock owners. However, if both incidence and net returns are considered it becomes apparent that the use of Isometamidium chloride treatment would be the most beneficial choice for farmers.

Studies such as these are difficult to undertake due to constraints in financial resources, distances to sampling sites, poor infrastructure, such as bad roads, the remoteness of the study sites, limited diagnostic facilities, illiteracy of farmers and the climate. These constraints can influence the quality of samples collected despite the best efforts of investigators. From a laboratory perspective quality control was conducted using an independent laboratory at the University of Zambia in another province to mitigate some of the effect on results that may have occurred. The purposive selection of farmers may have introduced some bias but there was little variability in farming practices and herd composition in the region of the study.

## Conclusions

The Cyfluthrin pour-on was the most effective vector control technique in reducing trypanosome incidence in cattle while treatment with Isometamidium chloride was most effective as a parasite control technique. These findings will help communities make better decisions in the choice of trypanosomiasis control methods based on their effect on trypanosome incidence. This will enable better use of the limited resources, which will in turn protect the livelihood of communities through reduced deaths thus improving food security.

## Supporting information

Mulenga et al. supplementary material Mulenga et al. supplementary material

## Data Availability

All data generated or analysed during this study are available in the James Cook University data repository.

## Supplementary material

The supplementary material for this article can be found at https://doi.org/10.1017/S0031182024001070.
