# Supplementary material for: An evaluation of African animal trypanosomiasis control strategies in remote communities of Eastern Zambia
Source: Parasitology. 2024 Oct 30;151(11):1269–76. doi: 10.1017/S0031182024001070 (PMC11894018; doi:10.1017/S0031182024001070)
Supplement: Mulenga et al. supplementary material [file S0031182024001070sup001.docx]

**SUPPLEMENTARY DATA**

1. **INFORMATION SHEET FOR LIVESTOCK FARMERS TO ENROLL ANIMALS IN STUDY**

PROJECT TITLE: **The control of Bovine and Human African Trypanosomiasis and role tsetse endosymbionts play in disease transmission in endemic areas of Zambia.**

| You have been purposively selected because of your farming experience to take part in a research project that aims at evaluating and identifying strategies and measures that are economically important in the control of African trypanosomiasis. The study will help in improving detection of African trypanosomiasis in domestic animals in endemic areas of Zambia. This will help the authorities make decisions when planning control programmes.  The study will also help in our knowledge of the role and importance different control strategies play in the control of Animal Trypanosomiasis so that we can identify what will be the most cost-effective way of controlling this disease in your region of Zambia. This could help make the control of the disease more affordable, reduce livestock deaths and improve rural livelihood in your region. The study is being conducted by **Gloria Mulenga** and will contribute to the attainment of a **Doctor of Philosophy Degree (PhD)** in **Epidemiology** at James Cook University in Australia. | |
| --- | --- |
| If you agree to be involved in the study, you will be invited to enrol your cattle in the study. With your consent, your enrolled cattle will be followed for a period of 12 months during which blood will be collected using capillary tubes from the ear veins. A cross section of goats if present will also be involved in the first month of the study only. All animals that will be found infected will be treated accordingly. Cattle enrolled will be ear tagged for easy identification. The study has potential to cause physical stress and injury to the animals during handling. Trained field animal health workers will be involved in the handling process to minimise the risks of injury. | |
| Taking part in this study is completely voluntary and you are free to withdraw from the study without penalty. | |
| The data from the study will be used in research publications and reports to be published by the principal investigator and other collaborators through **James Cook University, University of Pretoria, Zambian Government and University of Zambia**. You will not be identified in any way in these publications. | |
| If you have any questions about the study, please contact **– Gloria Mulenga (Principal Investigator) and Bruce Gummow (Supervisor).** | |
| **Principal Investigator:**  **Gloria Mulenga**  **Department of Veterinary Services**  **Ministry of Agriculture and Livestock**  **Republic of ZAMBIA**  **Phone: +61(07)4781 6903**  **Mobile:**  **Email: gloria.mulenga@my.jcu.edu.au** | **Australian Investigator:**  **Name: Bruce Gummow**  **College of Public Health, Medical and Veterinary Science**  **James Cook University, AUSTRALIA**  **Phone: +61(07)47814071**  **Mobile:**  **Email: bruce.gummow@jcu.edu.au** |

***If you have any concerns regarding the ethical conduct of the study, please contact:***

***Human Ethics, Research Office***

***James Cook University, Townsville, Qld, 4811***

***Phone: (07) 4781 5011 (***[***ethics@jcu.edu.au***](mailto:ethics@jcu.edu.au)***)***

1. **CONSENT FORM**

| **PRINCIPAL INVESTIGATOR**:  Gloria M. Mulenga |  | | | | |
| --- | --- | --- | --- | --- | --- |
| **PROJECT TITLE**: The control of bovine and human African trypanosomiasis and the role tsetse endosymbionts play in disease transmission in endemic areas of Zambia |  | | | | |
| INSTITUTION: James Cook University |  | | | | |
|  | | | | | |
| I understand the aim of this research study is **“To evaluate and identify strategies and measures that are economically important in the detection and control of African Trypanosomiasis in endemic areas of Zambia”.** I consent voluntarily to participate in this project, the details of which have been explained to me, and I have been provided with a written information sheet to keep. | | | | | |
| I understand that my participation will involve enrolling my cattle which will be followed for a period of 12 months during which blood will be collected using capillary tubes from the ear veins. All animals that will be found infected with trypanosomiasis will be treated accordingly.  Taking part in this study is completely voluntary and you are free to withdraw from the study without penalty. I agree that the researcher may use the results as described in the information sheet. | | | | | |
| I acknowledge that: | | | | | |
|  | | | | | |
| - Taking part in this study is voluntary and I am aware that I can stop taking part in it at any time without explanation or prejudice. | | | | | |
| - That any information I give will be kept strictly confidential/anonymous and that no names will be used to identify me with this study without my approval. | | | | | |
|  | | | | | |
| *(Please tick to indicate consent)* | | | | | |
| **I consent to be interviewed** | |  | **Yes** |  | **No** |
